# Complete sequences of the mitochondrial DNA of the *Petalonia binghamiae*

**DOI:** 10.1080/23802359.2017.1407688

**Published:** 2018-01-05

**Authors:** Yue Li, Na Liu, Hongxin Yin, Cui Liu, Lei Zhang, Yuemei Jin, Haiyang Wang, Shan Chi, Tao Liu

**Affiliations:** a Laboratory of Genetics and Breeding of Marine Organism, College of Marine Life Sciences, Ocean University of China, Qingdao, People’s Republic of China;; b Qingdao Haida BlueTek Biotechnology Co., LTD, Qingdao, People’s Republic of China

**Keywords:** Mitochondrial genome, *Petalonia binghamiae*, phylogenetic analysis

## Abstract

Here, the complete mitogenome of *Petalonia binghamiae* (J. Agardh) K. L. Vinogradova was determined and analyzed. The length of the complete *P. binghamiae* mitogenome was determined to be 37,460 bp. The mitogenome contains a set of 68 genes, including 35 protein-coding genes, 3 rRNA genes, 24 tRNA genes, 1 tRNA pseudogene, and 5 unidentified open reading frames (ORFs). The noncoding sequence constitutes 7.03% of the *P. binghamiae* mtDNA. Twenty-seven of the 35 (77.14%) protein-coding genes ended with a TAA stop codon, 6 (17.14%) with TAG, and 2 (5.71%) with TGA. All protein-coding genes in *P. binghamiae* were inferred to use the start codon ATG, except for *nad*11. The mitogenome phylogenetic analysis, based on 35 protein-coding genes, reveals that *P. binghamia* showed that *P. binghamiae* was clustered together with *P. fascia.* The complete mitochondrial genome sequence provided here would be useful for further understanding the evolution of *Petalonia*.

*Petalonia binghamiae* (J. Agardh) K. L. Vinogradova is an edible, economically important marine brown alga belonging to the family Scytosiphonaceae (Abbott and Hollenberg 1976). This species is found in the upper to mid-tidal zones of warm to temperate coastal areas throughout the Pacific Ocean and Indian Ocean, including along the coasts of North America, Korea, Japan, Pakistan, South Africa, Taiwan, and mainland China. The life history (Brophy and Murray 1989), morphological variability (Rhew and Boo 1991; Brophy 1995), chemical composition (Bano et al. 1987), and photosynthetic characteristics (Zou and Gao 2010) of this species have been studied both in the field and in culture, but only limited information is available about its genetics and systematics.

Here, we determined the complete mitogenome sequence of *P. binghamiae.* The genomic DNA of one *P. binghamiae* individual taken from a population located in eastern China (Nanji Island, Zhengjiang Province, 27°28′1′′N, 121°3′12′′E) was used for the genome sequencing. Specimen (accession number: 2016050053) was deposited in the Culture Collection of Seaweed at the Ocean University of China. Paired-end reads were sequenced using a Illumina’s HiSeq × Ten system (Illumina, San Diego, CA, USA). The genomic library was assembled using SOAPdenovo2 software (Luo et al. 2012). Transfer RNA genes were identified using the tRNAscan-SE Search Server (Schattner et al. 2005). Other regions of the mitogenome were annotated from the mitogenome of *P. fascia* using Geneious R10 (Biomatters Ltd., Auckland, New Zealand). Phylogenetic analysis of a set of 35 conserved protein-coding genes present in the 16 Phaeophyceae mitogenome was conducted. Maximum likelihood (ML) tree search and ML bootstrap analysis were performed using RaxML (Stamatakis 2006). Bootstrap probability values were run with 1000 replicates under the CpREV + G + I + F model.

The complete mitogenome of *P. binghamiae* (MF374731) consists of a circular DNA molecule with a length of 37,460 bp. The overall A + T content of the compete mitogenome is 65.6%. The mitogenome contains a set of 68 genes, including 35 protein-coding genes, 3 rRNA genes, 24 tRNA genes, 1 tRNA pseudogene, and 5 unidentified open reading frames (ORFs). No intron was observed in the mitochondrial coding regions. The lengths of three ribosomal RNA genes are 2717 bp (*rnl* rRNA), 1530 bp (*rns* rRNA), and 129 bp (*rrn5* rRNA). A total of 2481 bp of noncoding sequences (intergenic spacers) were present in the mitogenome. Thirty-five protein-coding genes were identified in *P. binghamiae*. Twenty-seven of the 35 (77.14%) protein-coding genes ended with a TAA stop codon, 6 (17.14%) with TAG, and 2 (5.71%) with TGA. All protein-coding genes in *P. binghamiae* were inferred to use the start codon ATG, except for *nad*11.

Maximum likelihood (ML) analyses showed that *P. binghamiae* was clustered together with *P. fascia* (Figure 1); moreover, five clades in the ML tree were identified, representing five different orders (Desmarestiales, Dictyotales, Ectocarpales, Fucales, and Laminariales) (Figure 1). The complete mitochondrial genome sequence provided here would be useful for further understanding the evolution of *Petalonia*.

**Figure 1.**
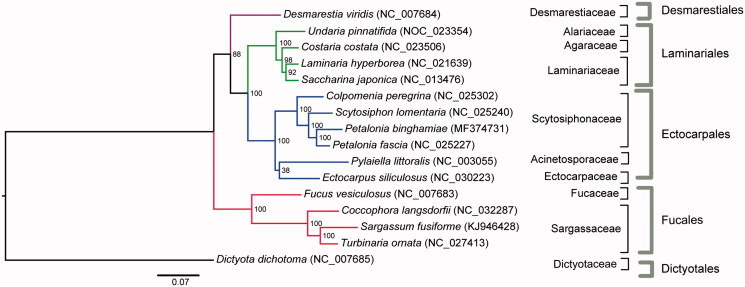
Phylogenetic tree (ML) of 16 Phaeophyceae species based on 35 mitochondrial protein-coding genes.
